# Low Antioxidant Glutathione Levels Lead to Longer Telomeres: A Sex-Specific Link to Longevity?

**DOI:** 10.1093/iob/obad034

**Published:** 2023-09-23

**Authors:** A A Romero-Haro, J Figuerola, C Alonso-Alvarez

**Affiliations:** Instituto de Investigación en Recursos Cinegéticos (IREC), CSIC-UCLM-JCCM, Ronda de Toledo 12, 13071Ciudad Real, 41092 Sevilla, Spain; Estación Biológica de Doñana—CSIC, Sevilla, 28029 Madrid, Spain; CIBER Epidemiología y Salud Pública (CIBERESP), Madrid, Spain; Evolutionary Ecology Department, National Museum of Natural Sciences (MNCN-CSIC), C/José Gutiérrez Abascal 2, 28006 Madrid, Spain; Instituto Pirenaico de Ecología (IPE-CSIC) Avda. Nuestra Señora de la Victoria, 16. 22700 Jaca, Huesca, Spain

## Abstract

Telomeres are repetitive DNA sequences at the end of chromosomes that protect them from degradation. They have been the focus of intense research because short telomeres would predict accelerated ageing and reduced longevity in vertebrates. Oxidative stress is considered a physiological driver of the telomere shortening and, consequently, short lifespan. Among molecules fighting against oxidative stress, glutathione is involved in many antioxidant pathways. Literature supports that oxidative stress may trigger a compensatory “hormetic” response increasing glutathione levels and telomere length. Here, we tested the link between total glutathione concentration and telomere length in captive birds (zebra finches; *Taeniopygia guttata*). Total glutathione levels were experimentally decreased during birds’ growth using a specific inhibitor of glutathione synthesis (buthionine sulfoximine; BSO). We monitored the birds’ reproductive performance in an outdoor aviary during the first month of life, and their longevity for almost 9 years. Among control individuals, erythrocyte glutathione levels during development positively predicted erythrocyte telomere length in adulthood. However, BSO-treated females, but not males, showed longer telomeres than control females in adulthood. This counterintuitive finding suggests that females mounted a compensatory response. Such compensation agrees with precedent findings in the same population where the BSO treatment increased growth and adult body mass in females but not males. BSO did not influence longevity or reproductive output in any sex. However, early glutathione levels and adult telomere length interactively predicted longevity only among control females. Those females with “naturally” low (non-manipulated) glutathione levels at the nestling age but capable of producing longer telomeres in adulthood seem to live longer. The results suggest that the capability to mount a hormetic response triggered by low early glutathione levels can improve fitness via telomere length. Overall, the results may indicate a sex-specific link between glutathione and telomere values. Telomerase activity and sexual steroids (estrogens) are good candidates to explain the sex-biased mechanism underlying the early-life impact of oxidative stress on adult telomere length.

## Abstract (es)

Spanish abstract

Los telómeros son secuencias repetitivas de ADN que se encuentran al final de los cromosomas protegiéndolos de la degradación. Los telómeros han sido foco de intensa investigación ya que tener telómeros cortos predice un envejecimiento acelerado y una longevidad reducida en vertebrados. El estrés oxidativo se considera causante del acortamiento de los telómeros y, consecuentemente, una corta esperanza de vida. Entre las moléculas que luchan contra el estrés oxidativo, el glutatión está relacionado con numerosas vías metabólicas de otros antioxidantes. La literatura defiende que el estrés oxidativo podría desencadenar una respuesta hormética compensatoria, aumentando los niveles de glutatión y la longitud de los telómeros. En este trabajo testamos la relación entre los niveles de glutatión y la longitud de los telómeros en aves en condiciones de cautividad (diamantes mandarines; *Taeniopygia guttata*). Los niveles de glutatión fueron reducidos experimentalmente durante el desarrollo de las aves usando un inhibidor específico de la síntesis de glutatión (Buthionina sufolximina; BSO). El desempeño reproductivo de estas aves fue monitoreado en un aviario exterior durante los primeros meses de vida y la longevidad durante casi nueve años. Entre los individuos controles, los niveles de glutatión durante el desarrollo predijeron positivamente la longitud de los telómeros a edad adulta. Sin embargo, las hembras tratadas con BSO, pero no los machos, mostraron telómeros más largos a edad adulta que las hembras controles. Este hallazgo aparentemente contradictorio sugiere que las hembras desarrollaron una respuesta compensatoria. Tal compensación concuerda con los hallazgos anteriores obtenidos en la misma población: las hembras tratadas con BSO vieron aumentado su peso corporal a edad adulta, resultado no encontrado entre los machos. El tratamiento con BSO no afectó la longevidad o el desempeño reproductivo de ningún sexo. Sin embargo, los niveles de glutatión durante el desarrollo y la longitud de los telómeros a edad adulta predijeron interactivamente la longevidad de las hembras controles. Aquellas hembras con niveles bajos “naturales” (no manipulados) de glutatión cuando eran pollos pero que pudieron producir telómeros más largos a edad adulta vivieron más. Este resultado sugiere que la capacidad de desarrollar una respuesta hormética desencadenada por niveles bajos de glutatión durante el desarrollo puede mejorar la eficacia biológica a través de la longitud de los telómeros. De manera general, los resultados indicarían una relación específica del sexo entre los valores de glutatión y de telómeros. La actividad de la enzima telomerasa y los esteroides sexuales (estrógenos) son buenos candidatos para explicar este mecanismo dependiente del sexo que impulsa el impacto del estrés oxidativo sufrido durante el desarrollo en la longitud de los telómeros de adultos.

## Introduction

In searching for proximate mechanisms explaining senescence and the evolution of life history traits, researchers from different fields have focused their interest on the length and dynamics of telomeres. Telomeres are highly repetitive non-coding DNA sequences at the end of eukaryotic chromosomes that protect the genetic material during cell division and safeguard genomic integrity and cell viability (Greider 1991; Blackburn 2001; Shay and Wright 2019; Srinivas et al. 2020). At the cellular level, telomeres shorten with each cell division cycle, reaching a point where the cell becomes unviable and dies (e.g., Allsopp et al. 1992; Kaul et al. 2012). At the individual level, there is increasing evidence that telomeres may also shorten throughout the lifetime in vertebrates (Remot et al. 2021). Long telomeres have been associated with phenotypic quality, reproductive performance, and low mortality risk (Le Vaillant et al. 2015; Wilbourn et al. 2018; Heidinger et al. 2021). Consequently, it has been suggested that telomeres might serve as a biomarker of individual quality and a predictor of longevity (e.g., Harley 1991; Olovnikov 1996; Heidinger et al. 2012; Tricola et al. 2018; Wang et al. 2018).

One mechanism pointed out as a relevant trigger of telomere attrition is oxidative stress (von Zglinicki 2002; Kawanishi and Oikawa 2004; Houben et al. 2008; Reichert and Stier 2017; Chatelain et al. 2020; Armstrong and Boonekamp 2023). Oxidative stress is the imbalance between the production rate of reactive oxygen and nitrogen species by cell metabolism and the state of the antioxidant machinery, leading to oxidative damage to main biomolecules, including DNA (e.g., Sies et al. 2017). Oxidative stress has recurrently been considered as a relevant promoter of ageing (Harman 1956; Beckman and Ames 1998; Barja 2014). A negative link between oxidative stress and telomere length was initially supported by experiments where pro-oxidative agents induced greater oxidative damage in telomeres than in other chromosome regions (e.g., Petersen et al. 1998; Oikawa and Kawanishi 1999; also Lin and Epel 2022 and references therein). A lower activity of intracellular antioxidant enzymes such as superoxide dismutase has also been linked to telomere attrition (Tarry-Adkins et al. 2006; Ahmed and Lingner 2018). Similarly, *in vitro* and *in vivo* experiments reported how antioxidants such as vitamins C and E can reduce telomere shortening (Furumoto et al. 1998; Kashino et al. 2003; Badas et al. 2015; Pineda-Pampliega et al. 2020). Overall, *in vivo* studies mainly support a negative association between oxidative stress and telomere length (see recent reviews in Reichert and Stier 2017; Armstrong and Boonekamp 2023).

In this framework, glutathione, one of the main intracellular antioxidants (e.g., Griffith and Meister 1985; Wu et al. 2004) also seems to be linked to telomere length variability. Total glutathione levels and telomere length were positively correlated in the blood of mice and humans (Stauffer et al. 2018; Yadav and Maurya 2022). Moreover, the *in vitro* administration of a glutathione derivative into marrow bone cells of rats increased the telomere length (Aminizadeh et al. 2016). Similarly, the administration of a blocker of the synthesis of glutathione (buthionine sulfoximine; BSO) decreased total glutathione levels, but also telomere length in different tissues of mammalian models under both *in vitro* (Kurz et al. 2004; Ozsarlak-Sozer et al. 2011, but see also Biroccio et al. 2003) and *in vivo* (Cattan et al. 2008) conditions.

In the present study, we explore the influence of low glutathione levels experienced during development on telomere length at adulthood and, subsequently, on key life-history traits (longevity and reproduction parameters). We aimed to understand if a potential interaction between early glutathione levels and adult telomere length influences fitness traits. For that purpose, we manipulated glutathione levels *in vivo* in captive birds, i.e., zebra finches (*Taeniopygia guttata*), using a BSO treatment. We previously described how an *in vivo* administration of BSO decreased erythrocyte total glutathione concentrations and plasma levels of hydrophilic antioxidants in zebra finch nestlings (Romero-Haro and Alonso-Alvarez 2015). Interestingly, the BSO treatment exerted stronger effects later in life than early (Romero-Haro and Alonso-Alvarez 2015). In adulthood, the BSO-treated birds showed greater lipid oxidative damage than controls, and BSO-treated females reported a lower erythrocyte resistance to oxidative stress-induced hemolysis compared to control females (Romero-Haro and Alonso-Alvarez 2015). Moreover, females also gained more body mass and attained a higher size-corrected body mass in 40 days and after sexual maturity than controls, but not earlier, supporting a compensatory delayed response (“hormesis”; e.g., Calabrese et al. 2007) that would have modified the developmental trajectories and adult phenotypes (Romero-Haro and Alonso-Alvarez 2015).

Here, we predict that if glutathione protects telomeres from oxidative stress-induced telomere attrition, a positive correlation between telomere length and glutathione concentration in erythrocytes of control individuals should emerge (Stauffer et al. 2018; Yadav and Maurya 2022). Similarly, we predict that since BSO-treated zebra finches endured higher oxidative stress than controls, they should have also developed shorter telomeres in their erythrocytes and, consequently, reduced longevity and reproductive outcomes (see Heidinger et al. 2021). Importantly, considering the cited previous evidence on the same population of birds (i.e., Romero-Haro and Alonso-Alvarez 2015), we also foresee that BSO treatment would mostly affect telomere length in adulthood instead of shortly after administration, effects being also more evident among females (see above). Alternatively, we may also consider the possibility of a compensatory response on telomere length, such as that detected in the case of the body mass of females.

## Methods

### Experimental manipulation of glutathione levels during development

The present study is part of a long-term experiment testing the impact of the experimental decrease of the levels of an important intracellular antioxidant (glutathione) during development in the expression of life-history traits of a captive population of zebra finches (Romero-Haro and Alonso-Alvarez 2015, 2020; Romero-Haro et al. 2015; Romero-Haro et al. 2016). Eighty randomly formed zebra finch pairs (F0 birds) obtained from three different Spanish commercial providers were housed in breeding cages (0.6 m  × 0.4 m  × 0.4 m) with a nest box, water, and food *ad libitum* (housing details in Romero-Haro and Alonso-Alvarez 2015). The pairs were allowed to breed for about 5 months, and their reproduction was monitored every 2 days (hatchlings produced from October 21, 2011 to March 31, 2012). A total of 12 pairs did not breed, 22 pairs reproduced once, 22 pairs reproduced twice, 21 pairs reproduced three times, and 3 pairs produced four broods.

An experimental treatment was applied to chicks (F1) produced by the breeding pairs (F0) when they reached a minimum weight of 3 g (mean ± SE: 4.82 g ± 0.03). Half of the nestlings in a brood were assigned to the treatment receiving DL-buthionine-S, R-sulfoximine (BSO; Sigma, ref. B2640) diluted in sterilized saline solution (0.90% w/v of NaCl; BSO individuals). The other half was assigned to the control group that only received sterilized saline. The BSO solution was 50 mg BSO in 1 mL of saline. A volume of 0.06 mL of this BSO solution (BSO individuals) or same volume of saline (controls) was subcutaneously injected into the chicks’ backs every 2 days from 6 to 12 days old (i.e., four injections). Total glutathione (tGSH) in erythrocytes was determined in 14-day-old nestlings (see analytic method below).

The election of that BSO dose was based on two previous studies. First, several dosages were tested (0, 0.007, 0.292, and 0.579 g/Kg body mass/day; total: 0, 0.4, 16.4, and 32.4 total mg per bird, respectively) in great tit (*Parus major*) nestlings (*n* = 53, 51, 55, and 45, respectively), detecting a significant decline in erythrocyte total glutathione levels at the two highest dosages (Galvan and Alonso-Alvarez 2008). From these findings, a pilot experiment was performed in zebra finch nestlings with similar age and body masses to those described in the definitive experiment (above). Birds received BSO dosages similar to the range of those used in great tits (0, 0.094, 0.188, and 0.375 g/Kg body mass/day; total: 0, 3, 6, and 12 total mg per bird). These dosages consider body mass differences between both species. A total of 26 nestlings from 10 broods were used. Twenty-three birds provided enough blood volume for the analyses (i.e., *n *= 6, 6, 6, and 5, respectively). The highest BSO dosage (i.e., 12 mg) was chosen based on the mean erythrocyte total glutathione levels in 14-day-old nestlings (means ± SD; control: 3.76 ± 0.74, 3 mg; 3.23 ± 0.22, 6 mg: 3.72 ± 0.59, and 12 mg: 3.28 ± 0.24) and also considering the significant differences in great tits (above). Subsequently, in the definitive experiment, BSO birds treated with the cited dosage showed significantly lower total glutathione levels than controls (Romero-Haro and Alonso-Alvarez 2015; see also below).

The experimental chronogram is shown in the Supplementary Material (Fig. S1). We randomly assigned one treatment to the heaviest chick in a brood and then alternated the treatment assignation among the siblings (e.g., control, BSO, control, BSO). Twelve nestlings were excluded as they erroneously received a mix of the two treatments. Finally, 206 BSO-treated and 203 control nestlings were obtained. For each nestling, a blood sample was collected from the brachial vein 8 days after the beginning of the injection period (Fig. S1; mean age: 14 days). Males and females were separately housed in different rooms (2.80 m × 3 m × 2.50 m) when they reached approximately 40 days of age (mean ± SE: 39 days ± 0.12), i.e., when all the birds were independent of the parental care (details in Romero-Haro and Alonso-Alvarez 2015). In adulthood, a second blood sample was collected from the jugular vein (mean ± SE: 97.8 days ± 0.84). For simplicity, we use 40 and 100 days to show the mean age for independence and adulthood, respectively. The blood was stored at 4°C and centrifuged within 4 h. The plasma was removed, and the buffy coat was discarded. Plasma and erythrocyte fractions were separately stored at −80°C. Each individual's tarsus length and body mass were measured on the day of the first injection, first blood extraction, independence, and adulthood.

### Monitoring of the reproduction and lifespan

A random subsample of the F1 birds was released in a large outdoor aviary when they reached a minimum age of 110 days (mean ± SE: 137 days ± 1.52 days). This subsample represents the 65% of individuals of the F1 generation who reached the releasing age (i.e. 183 from 283 birds). The subsample was statistically balanced in terms of treatment and sex (easily defined by sexual dimorphic traits and also molecularly; Romero-Haro and Alonso-Alvarez 2015): 49 control males, 45 control females, 39 BSO males, and 50 BSO females; treatment X sex balanced: *χ*^2^ = 1.26, *P* = 0.261). In this subsample, BSO-treated birds also showed lower glutathione levels at 14 days of age than controls (*P* <0.001, Cohen's *d* = 0.636). The birds were released in two events separated by 40 days, the sex and treatments being also balanced within each event (all *χ*^2^ tests: *P* >0.40).

The outdoor aviary had a 74.4 m^2^ surface (12 m long × 6.20 m width) and was 3 m high. The number of birds in the cited aviary volume (i.e., 1.20 m^3^ per bird) was chosen to avoid the stress produced by higher densities (Poot et al. 2012). The birds were allowed to mate freely and breed in this aviary for 7 months from the release time (more details in Romero-Haro et al. 2016; Romero-Haro and Alonso-Alvarez 2020). The reproductive output was monitored every 2 days. The aviary included 194 nest boxes (14 cm × 12 cm  × 16 cm) to allow reproduction, i.e., doubling the number of potential couples. The birds received *ad libitum* food and water (more details in Romero-Haro et al. 2016). The birds were involved in a cross-fostering experiment that also enlarged or reduced brood sizes to increase or decrease the parental reproductive effort respectively. The manipulation of reproduction (increased or decreased effort) or its interaction with sex or early treatment did not significantly influence the longevity of individuals (all *P*-values > 0.240; unpublished results). The early treatment (BSO vs. control) did not affect the proportion of eggs hatched in the broods produced in the outdoor aviary (Binomial GLIMMIX in SAS with couple identity as a random coefficient term: father BSO treatment: *F*_1,66_ = 0.01, *P* = 0.956; mother BSO treatment: *F*_1,66 _= 0.02, *P* = 0.886; interaction: *F*_1,66 _= 0.20, *P* = 0.656), discarding differential embryo mortality (see details in Romero-Haro and Alonso-Alvarez 2020).

After the reproductive period, males and females were separated and each sex was placed in one-half of the aviary (half of the cited volume, similar bird density). The lifespan was then monitored each 2–3 days throughout more than 8 years (2012–2020) until the death of the oldest bird (at 8 years and 8 months of age). The regular monitoring consisted of retrieving and identifying corpses during each visit or, instead, checking the list of birds during the sampling events performed throughout the cited 8-year period. Unfortunately, some corpses disappeared due to ants entering the aviary and quickly removing the bodies. These missing birds could only be identified when capturing all the birds during sampling events. In that case, we used the last date when the bird was found alive as a proxy of longevity, censoring that specific data in the Cox models (below). Moreover, some birds died of causes unrelated to ageing (accidents when flying or during sampling, or predation by snakes entering the facilities). In this case, we calculated the longevity value by using the date when the animal was found dead, also censoring this data in the models. Finally, the longevity data of 29% of individuals were censored. The comparison of occurrence or absence of censored data between the two sexes, treatments or the four sex X treatment combinations never provided significant biases (*χ*^2^ tests *P*-values >0.27).

### Total glutathione levels in red blood cells

The total glutathione concentration was quantified following Griffith's (1980) method with modifications (Romero-Haro and Alonso-Alvarez 2014). Briefly, erythrocytes were thawed and immediately diluted (1:10 w/v) and homogenized in a stock buffer (0.01M PBS and 0.02M EDTA), working on ice to avoid oxidation. Three working solutions in the same stock buffer were created as follows: 0.3 mM NADPH (solution I), 6 mM DTNB (solution II), and 50 units of the enzyme glutathione reductase mL^−1^ (solution III). An aliquot (0.2 mL) of homogenate of blood cells with 0.2 mL of diluted trichloroacetic acid (10% in H_2_O) was vortexed 5 s three times: after dilution and at 5 and 10 min. In the interim, samples were protected from light and refrigerated to prevent oxidation. The mixture was then centrifuged (1125 *g* for 15 min at 6°C) and the supernatant removed. Subsequent steps were made in an automated spectrophotometer (A25-Autoanalyzer, Biosystems). Solutions I and II were mixed at a ratio of 7:1 v/v, respectively. An amount of 160 μL of this new mixture was automatically added to 40 μL of the sample (i.e., supernatant) in a cuvette. Then, 20 μL of solution III was added after 15 s, and the absorbance at 405 nm was monitored after 30 and 60 s. The change in absorbance was used to determine total glutathione levels (tGSH) by comparing the output with the results from a standard curve generated by serial dilution of glutathione from 1to 0.031 mM. Results are given in mM/g of pellet. Twenty-eight samples were measured in duplicate, obtaining a high Lessells’ repeatability (*R *= 0.95, *P *< 0.001). One sample was missing during handling, so there is not glutathione level for it.

### Telomere length analyses

The telomere length of those individuals released in the outdoor aviary was measured from erythrocyte pellet samples obtained at 14 and 100 days of age, stored at −80°C without buffer. All these samples endured the same single freeze-thaw cycle and the same period frozen at −80°C. DNA was extracted using an in-house extraction protocol based on magnetic beads (see Supplementary Material for a detailed protocol) and DNA was quantified using NanoDrop (Thermo Fisher Scientific). We estimated relative telomere length with the quantitative polymerase chain reaction (qPCR) assay and primers described by Criscuolo et al. (2009). qPCR provides an estimate of the amount of telomere sequence present in relation to a non-telomeric reference sequence. In this case, we used the sequence of the single-copy gene glyceraldehyde-3-phosphate dehydrogenase (GAPDH). Real-time amplification of telomere and GAPDH were done in different plates (Roche), running in duplicate in each plate a reference sample, diluted at concentrations of 40, 10, 2.5, and 0.66 ng/µL of DNA per well. Each reaction to measure telomere or GAPDH for the test samples used 2 µL of DNA at a concentration of 10 ng/µL, sets of primers Tel1b/Tel2b or GAPDH-F/GAPDH-R in a final volume of 20 µL containing 10 µL of Fast Start Universal SYBR Green Brilliant Master (Roche, Diagnostics GmbH, Mannheim, Germany) and 1 µL of bovine serum albumin (20 mg/mL). Bovine serum albumin was added to enhance the PCR amplification yield.

We performed qPCRs in a LightCycler 480 Real-Time PCR (RT-PCR) System (Roche), with PCR conditions for the telomere starting with 10 min at 95°C, followed by 30 cycles of 1 min at 56°C and 1 min at 56°C and 1 min at 95°C. Conditions for GAPDH were 10 min at 95°C, 40 cycles of 1 min at 60°C, and 1 min at 95°C. Melting curve plots were examined to confirm the specificity of the amplified products. Under optimal conditions, each qPCR cycle is expected to duplicate the DNA fragment amount, what corresponds to an efficiency of 2 in an exponential scale and of 100% when expressed as percentages. The efficiency of each PCR ranged between 1.864 and 2.030 for telomere (mean ± SD = 1.937 ± 0.038) and 1.879 and 2.035 for GAPDH (mean ± SD = 1.962 ± 0.049). Slopes for calibration curves for telomere varied between −3.698 and −3.252 (mean ± SD = −3.487 ± 0.103) and between −3.649 and −3.240 for GAPDH (mean ± SD = −3.423 ± 0.128). The coefficients of variation of the quantification cycle (Cq) for reactions from the same sample were lower than 5% in all samples, and all Cqs were measured in the linear dynamic range. Interplate CV was 1.51% for GAPDH and 2.82% for telomere, and intraplate CV was 0.60% for GAPDH and 1.57% for telomere. Similarly, repeatability across plates was 97% (*P* <0.001) for GAPDH and 98% (*P* <0.001) for telomere. To account for plate variations in efficiency, we calculated relative telomere length as normalized relative quantities (NRQs) following Hellemans et al. (2007). Briefly, Cq of both replicates was averaged. NRQs were calculated as the exponential of the plate efficiency to the difference between the Cq of the control sample and the average Cq of the sample of interest. Finally, relative telomere length was calculated as the ratio of telomere/GAPDH-NRQs.

The qPCR method measures terminal and interstitial telomeric repeats (Nakagawa et al. 2004). Although interstitial telomeric repeats vary between and within species (Foote et al. 2013), the amount does not change with individuals’ age (Delany et al. 2003). A precedent study in the same avian species showed that the telomere length measured from frozen blood pellets using qPCR or assessed on whole fresh blood by the terminal restriction fragment method were highly correlated (*R* = 0.87, *n* = 26; i.e., Criscuolo et al. 2009). The volume of the pellet used for the analyses (means ± SD = 45.4 ± 11.7 and 83.1 ± 18.2 mg for nestlings and adults, respectively) did not significantly correlate to telomere length measures at adult or nestling ages (Spearman or Pearson's *R* < 0.1 and *P* > 0.17).

Telomere length data could not be obtained for eight chicks and seven adult samples due to missing samples, insufficient sample volume or failed qPCR. For statistical analyses, telomere length values were sqrt-transformed to fulfil normality requirements and *z*-transformed (standardized; a distribution with a mean equal 0 and standard deviation equal 1) to allow comparisons between studies (Verhulst 2020).

### Statistical analyses

Firstly, to estimate the within-individual consistency of telomere length measurements between 14 and 100 days of life, we followed the procedure described in (Karkkainen et al. 2022). This repeatability coefficient consists of an intra-class correlation comparing the between-individual variance (by including the identity of individuals as a random factor) with the total variance. The life stage (fledging 14 days/adult 100 days) was included as a fixed effect. Confidence intervals (95% CI) of the repeatability coefficient were estimated running 1000 bootstraps. Repeatabilities including sex and early treatment as fixed effects are shown in the Supplementary Material (Table S1).

Subsequently, we aimed to test the predictions formulated at the end of the Introduction section. This was made by a linear mixed model testing the influence of the individuals’ sex, BSO treatment and 14-day-old total glutathione levels in erythrocytes (i.e., three explanatory terms) on telomere length at adulthood (100 days; i.e., the dependent variable). Moreover, we tested two- and three-level interactions as additional explanatory terms to explore potential connections between these parameters. The identity of the brood nested into the identity of the cage where the birds were raised and the laboratory session (plate) were included as random effects to account for the non-independence of biological siblings and among-laboratory session variation, respectively. Alternative models that included the body mass and age, the body mass increase from the beginning of the treatment until adulthood or the telomere length at 14 days as additional explanatory variables are shown in the Supplementary Material (Table S2). The models mentioned above, including telomere length at 14 days as the dependent variable instead of telomere length at 100 days, are shown in Table S3. A repeated-measurement mixed effect model reporting similar results is shown in Table S4.

After verifying that the early treatment, sex, and glutathione levels at 14 days affected telomere length at adulthood (previous model), we tested for any influence of the adult telomere length on individuals’ fitness: reproductive performance and longevity. In all the models, the early treatment, sex, and telomere length at 100 days and their three-level interaction were included as explanatory variables, and the ID of the brood nested into the ID of the origin cage as a random factor. For reproductive performance, the effects on latency to breed, number of eggs, and hatching success of the first reproductive event were tested. These reproductive variables were chosen because they cannot be affected by the brood size manipulation included in the long-term study of this population (i.e., Romero-Haro et al. 2016). Regarding this, we considered that the inclusion of the breeding manipulation factor would have reduced the statistical power of the models. To test the latency until the first reproduction, we ran a binomial generalized mixed effects model. To test the number of eggs or the hatching success of the first clutch, we used generalized mixed effect models with Poisson distribution. We ran mixed-effects Cox models for individuals’ longevity. This longevity Cox model was also ran including telomere length at 14 days instead of 100 days measurement. Note that breeding effort manipulation did not affect longevity (see above). The releasing event identity was considered as a fixed factor in all the fitness analyses, being always non-significant and finally not included in the models, except for latency to breed where it showed a trend to significance (Table S6).

When testing physiological variables as explanatory variables in the models (e.g., the 14-day-old glutathione level on the adult telomere length model and the telomere length at 100 days on the fitness models), we included the residuals from models controlling by random factors. This approach is needed since random factors control the dependent variable and not the explanatory variables included in the model. So, residuals were obtained from mixed-effect models with glutathione levels or telomere length as dependent variables and the brood identity nested into the cage identity and the laboratory session as random effects. In the model used to obtain the glutathione residuals, we also included the treatment factor as an explanatory term because the dependent variable was the target of the manipulation, inducing a direct and strong effect on its variability (Romero-Haro and Alonso-Alvarez 2015). In any event, similar findings were attained when using glutathione residuals calculated without the treatment factor.

All statistical analyses were performed in *R* version 4.2.0. (R Core Team 2021). Within-individual repeatability was obtained with the function rpt of the package *rptR* (v. 0.9.22.) (Stoffel et al. 2017). The *R* package *lme4* (v. 1.1.32) (Bates et al. 2015) was used to perform (generalized) mixed effect models and *lsmean*s (v. 2.30.0) (Lenth 2016) for the pairwise *post hoc* comparisons. The *coxme* (v. 2.2.18.1) and *survival* (v. 3.3.1.) packages were used for mixed-effect Cox models (Therneau et al. 2003). The predictors’ significance was obtained with analysis of variance function from the *car* package (v. 3.1.1.) to perform likelihood-ratio tests for mixed effects models and generalized mixed effects models and Wald *χ^2^* test for cox models (Fox and Weisberg 2019). Normality and homoscedasticity assumptions of residuals were met.

## Results

### Repeatability of telomere length measurements

Erythrocyte telomere length measured at 14 and 100 days showed within-individual consistency (controlled by life stage (fledging 14 days/adult 100 days): *R* = 0.435; SE = 0.064; CI = 0.301–0.558, *P* < 0.001). The sex or early treatment did not substantially change the *R*-value (see Table S1).

### Influence of the early treatment and 14-day-old glutathione levels on adult telomere length

**Fig. 1:**
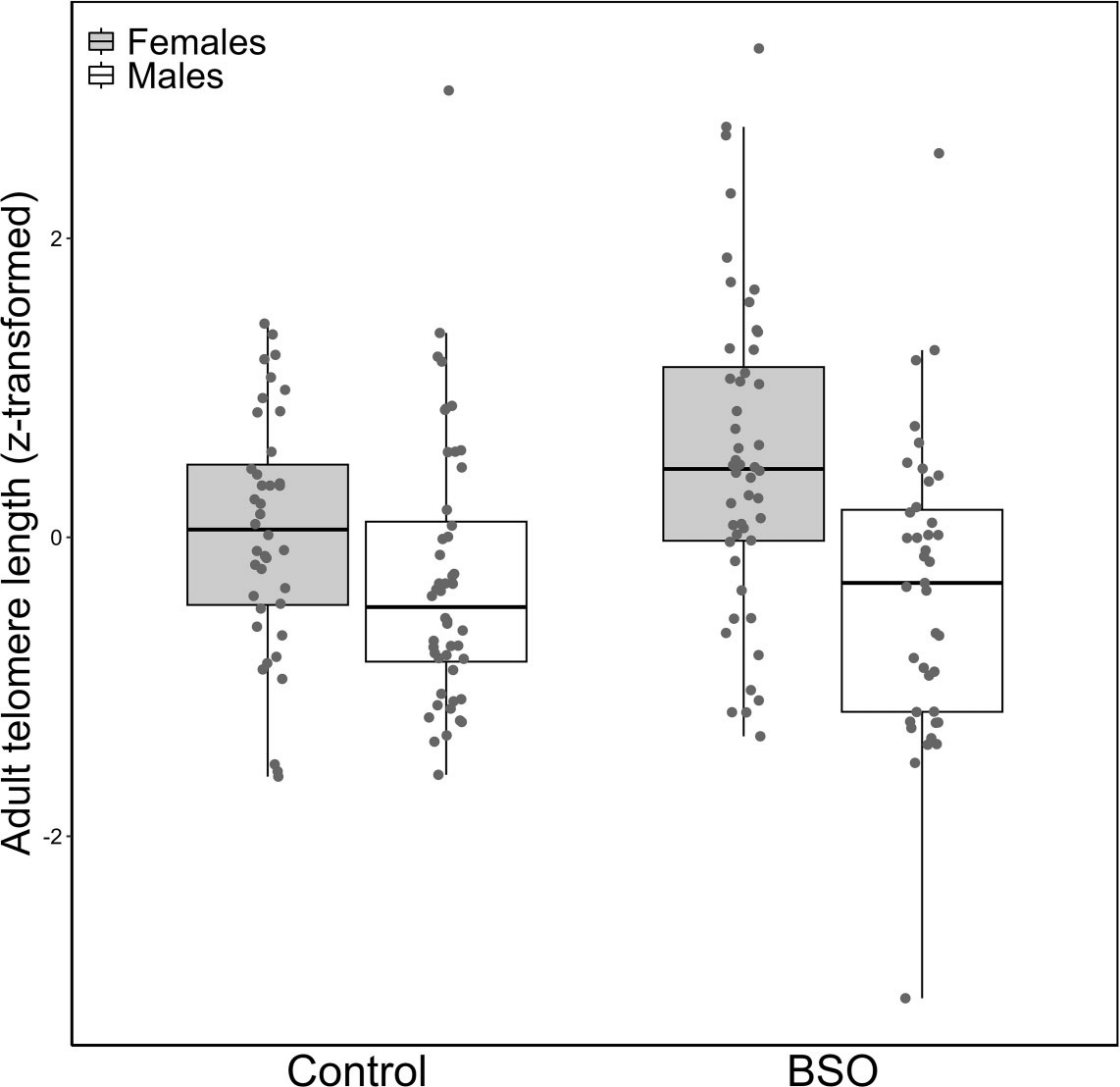
Telomere length at 100 days among Control and BSO individuals. Telomere length values (T/S ratio) were sqrt- and *z*-transformed (standardized). Horizontal lines in boxplots represent means and interquartile ranges. Individual data points are shown. Results do not change by removing the shortest telomere length in one BSO male (see Table S5 and Fig. S3).

**Fig. 2:**
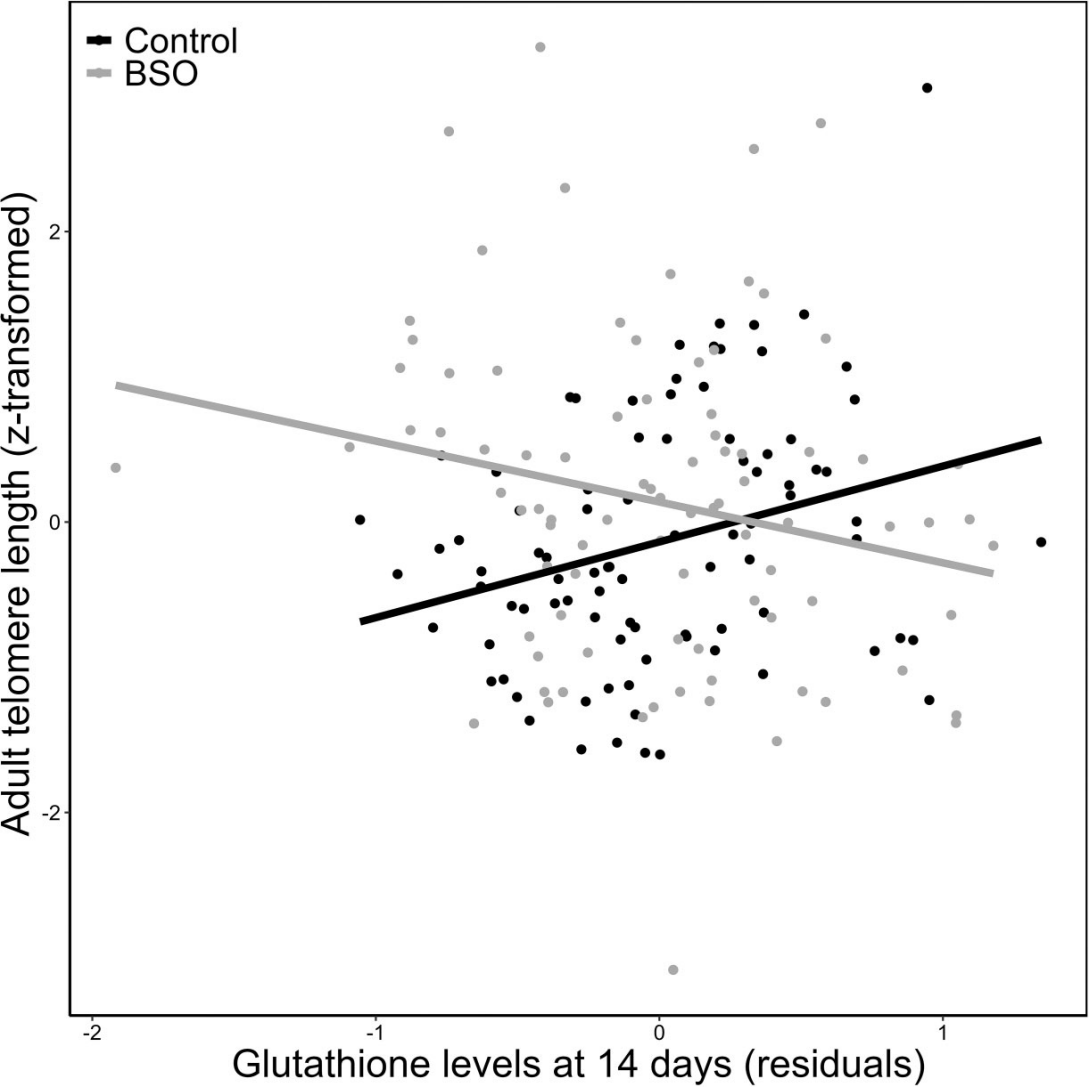
Association between telomere length at 100 days old and glutathione levels at 14 days old in control (in black) and BSO (in gray) individuals. Glutathione levels are the residuals obtained from mixed effect models with the ID of the brood nested into the ID of the origin cage and the laboratory session as random effects and the early treatment as an explanatory variable. Levels of glutathione at 14 days were experimentally decreased in BSO individuals. Telomere length values (T/S ratio) were sqrt- and *z*-transformed (standardized). Note that association is significant among control individuals and non-significant among BSO individuals. Results are similar when removing the lowest value of telomere length (see Table S5 and Fig. S4). In Fig. S5, two different panels for control and BSO individuals are shown.

Telomere length at adulthood (at 100 days of age, i.e., 86 days after the treatment) was affected by the interaction between the early treatment and sex (Table 1, Fig. 1). *Post hoc* contrast revealed that BSO females had longer telomeres than control females (mean ± 1SE: 0.351 ± 0.251 and 0.036 ± 0.257, respectively; *t* = 2.173, *df* = 138, *P* = 0.032; Fig. 1), whereas the telomere length of BSO and control males did not differ (−0.050 ± 0.258 and 0.122 ± 0.255, respectively; *t* = −1.184, *df* = 143, *P *= 0.238; Fig. 1). Similarly, while BSO females had longer telomeres than BSO males (*t* = 2.462, *df* = 159, *P* = 0.015; Fig. 1), telomere length of control males and females did not differ (*t* = −0.522, *df* = 157, *P* = 0.602; Fig. 1). The early treatment also affected the association between glutathione levels at 14 days (i.e., the manipulated variable) and telomere length at 100 days (treatment X early glutathione value interaction in Table 1; see also Fig. 2). In BSO-treated individuals, the glutathione levels during development showed a trend to a significant negative correlation to adult telomere length at 100 days (Estimate = −0.294, SE = 0.151, *χ*^2^ = 3.805, *P* = 0.051; see also Fig. 2 and Fig. S4 and S5B). Instead, early glutathione levels of control birds were significantly positively correlated to adult telomere length (Estimate = 0.324, SE = 0.132, *χ*^2^ = 6.039, *P* = 0.014; Fig. 2 and Fig. S4 and S5A). The telomere length at 14 days was significantly related to telomere length at 100 days, but this did not affect the main results explained above (Table S2). The body mass, age, or body mass increase since the beginning of the treatment did not affect the length of telomeres at 100 days (Table S2). Similarly, telomere length at 14 days (i.e., 2 days after the treatment) was not affected by the sex, treatment, glutathione levels at 14 days, body mass, age, or body mass increase (Table S3).

**Table 1:** Effects of the early treatment, sex, levels of glutathione at 14 days old (the manipulated variable) and their three-level interaction on telomere length at adulthood. Significant p-values are indicated in bold.

Explanatory variables	Estimate	SE	*χ^2^*	*df*	*P*
Intercept	0.351	0.251			
Treatment (control)	−0.315	0.144	0.625	1	0.429
Sex (males)	−0.401	0.161	1.789	1	0.181
14 days Glutathione	−0.414	0.169	0.023	1	0.879
Treatment (control) × Sex (males)	0.487	0.206	5.744	1	**0.017**
Treatment (control) × 14 days glutathione	1.031	0.275	11.807	1	**<0.001**
Sex (males) × 14 days glutathione	0.332	0.266	0.009	1	0.925
Treatment (control) × Sex (males) × 14 days glutathione	−0.740	0.418	3.127	1	0.077
**Random factors**	**Variance**	**SE**			
Cage ID (brood ID)	0.106	0.326			
Plate	0.516	0.718			
Residuals	0.377	0.614			

### The influence of the early treatment and telomere length at 100 days on fitness

Neither early treatment, sex nor telomere length at 100 days influenced the latency to breed, the clutch size, or the hatching success of the first reproductive event (all *P*-values >0.077; Table S6). Similarly, the early treatment, sex, or telomere length at 100 days or their interaction did not predict longevity (all *P*-values >0.127; Table S7). Moreover, telomere length at 14 days or its interactions with early treatment and sex did not predict longevity either (all *P*-values >0.097; Table S7).

### The influence of the early treatment, glutathione levels at 14 days, and telomere length at 100 days on longevity

**Fig. 3:**
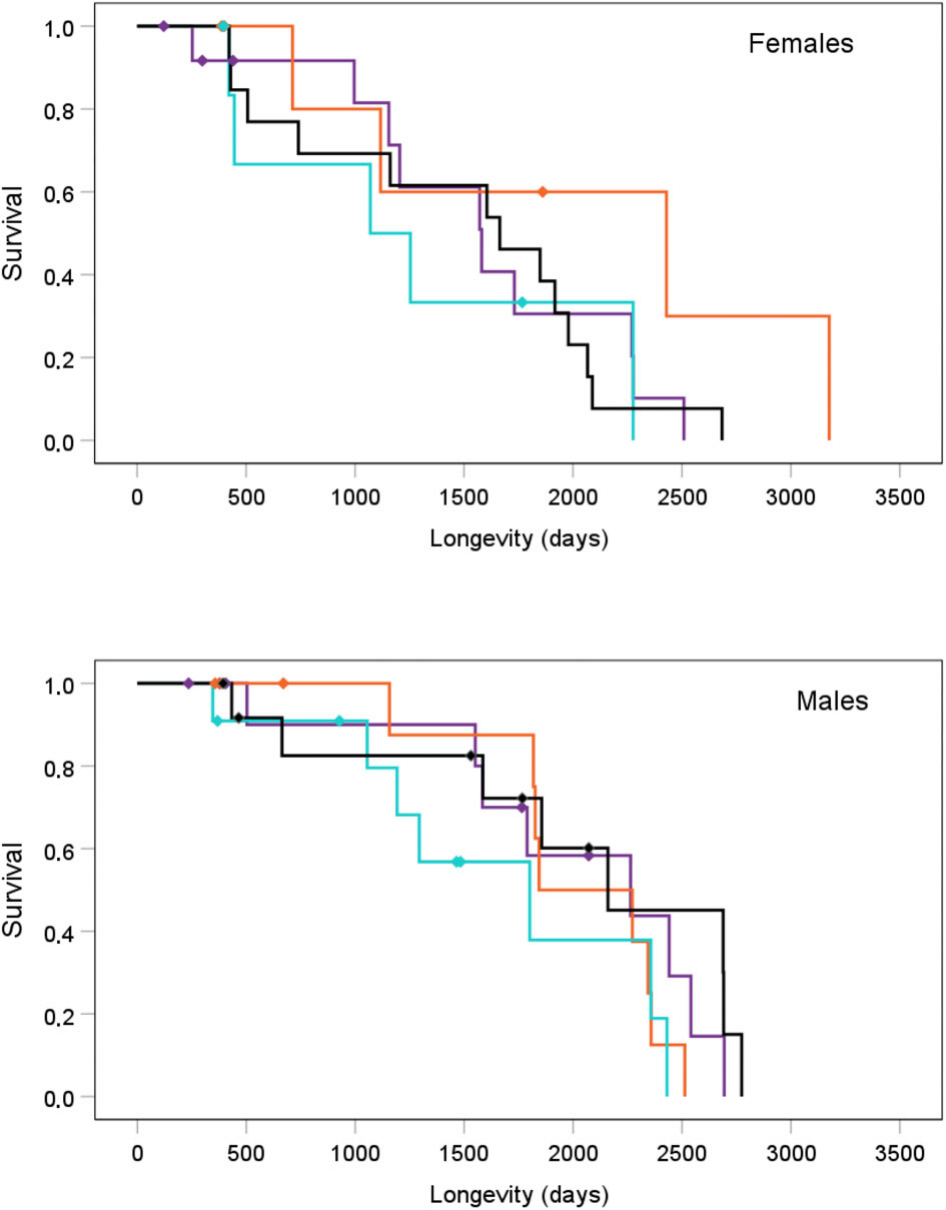
Cumulative survival of non-manipulated control female (above) and male (below) zebra finches. The sample was divided by the median of the residuals of glutathione at 14 days and adult telomere length (at 100 days) to illustrate the effect of the interaction between the two covariates. Black line: birds with telomere length and early glutathione values higher than their medians. Purple line: birds with telomere length and early glutathione values lower than their medians. Blue line: birds with telomere length and early glutathione values shorter and higher than their medians, respectively. Orange line: birds with telomere length and early glutathione values longer and lower than their medians, respectively. Birds with values equal to the mean were assigned to the lower than the median categories. Dots indicate the presence of censored data.

Since glutathione levels during development significantly predicted telomere length at adulthood in control individuals only (Fig. 2), the interactive effect of these two physiological variables on longevity was tested in that group of birds (see also Supplementary Material). In this order, a mixed-effects Cox model, including sex, glutathione levels at 14 days old, adult telomere length, and their interactions, was performed, again including brood identity nested into cage identity as a random factor. The longevity was indeed affected by the interaction between the explanatory terms (Sex [Males]  × 14 days Glutathione × Adult telomere length: *β* = −3.337, SE = 1.251, *χ*^2^ = 7.112, *P* = 0.008; also Table S8). Thus, the longevity of control females was significantly influenced by the interaction between early glutathione levels and adult telomere length (*β* = 2.200, SE = 1.038, *χ*^2^ = 4.497, *P *= 0.034). In contrast, that influence was not detected among control males (*β =* −1.096, SE = 0.773, *χ*^2^ = 2.007, *P* = 0.157). Figure 3 illustrates that effect and indicates that lifespan becomes longer in those control females with low early glutathione levels and long adult telomeres.

## Discussion

By experimentally decreasing the glutathione synthesis during early development, we tested the influence of this antioxidant on telomere length at adulthood and, subsequently, on the reproductive performance and longevity of captive zebra finches. Our correlational results among control individuals support a positive relationship between nestlings’ glutathione levels and adult telomere length. However, the experimental inhibition of glutathione production during development did not reduce erythrocyte telomere length as initially expected (e.g., Cattan et al. 2008). Quite the contrary, BSO-treated females (but not males) showed longer telomeres than control females in adulthood. Below, we interpret this as the consequence of a compensatory response to a harsh early environment (high oxidative stress) in that sex. The telomere length of adults was not directly related to any of the assessed fitness-related traits (longevity or several reproductive parameters). However, the positive correlation between early-age glutathione levels and adult telomere length in controls predicted female longevity. Adult control females with long telomeres lived longer if they had previously experienced low glutathione levels during early growth. Overall, our findings reveal that the association between glutathione levels and telomere length and its potential fitness consequences depended on the early development environment and the sex of the bird.

Our initial prediction that the experimental glutathione decrease should lead to shorter telomeres was based on the positive association between total glutathione levels and telomere length described in correlational and experimental studies in mammalian models (Kurz et al. 2004; Cattan et al. 2008; Ozsarlak-Sozer et al. 2011; Aminizadeh et al. 2016; Stauffer et al. 2018; Yadav and Maurya 2022). However, such a link is not significant in most of the few correlational avian studies (nestling jackdaws, *Corvus monedula*: Boonekamp et al. 2017; nestling flycatchers, *Fidecula hypoleuca*: Lopez-Arrabe et al. 2018; embryo great tits, *P. major*: Watson et al. 2018b, Pearson's *R* = −0.27, *P* = 0.145, from the public dataset, see also Watson et al. 2018a). Only in nestlings of spotless starlings (*Sturnus unicolor*), erythrocyte values of both physiological variables reported a significant but negative correlation among females, but not among males (Pearson's *R* = −0.21, *P* = 0.02, *n* = 128 and *R *= 0.05, *P *= 0.58, *n *= 132, respectively; from the public dataset; see Gil et al. 2018, 2019), supporting the view that females have a different antioxidant metabolism involving that relationship (see below).

Contrary to our initial prediction of a telomere reduction among BSO-treated birds, the delayed effect of the early BSO treatment on females’ telomere length suggests a compensatory (hormetic) response to early oxidative stress, matching some of our previous findings in females in the same captive population (Romero-Haro and Alonso-Alvarez 2015). That is, adult females (not males) that were treated with BSO during development reached a higher body mass than adult control females, attained a higher size-corrected body mass (often defined as “body condition index”; Labocha and Hayes 2012) and produced redder bills (a sexually selected ornament: Burley 1986; Simons et al. 2012) than controls (see Romero-Haro and Alonso-Alvarez 2015). Similarly, the results of telomere dynamics would agree with this. Although the interaction between life stage, sex, and treatment did only show a (non-significant) trend to significance (*P* = 0.085; i.e., Table S4). That trend was due to an apparent telomere elongation from 14 to 100 days of age in BSO-treated females compared to other groups that did not report any variation along with age (Fig. S2). We could argue that this pattern was due to hormesis, which has been associated with triggering mechanisms involving oxidative stress and glutathione-related redox signals (reviews in Costantini 2014; Calabrese and Kozumbo 2021). Regarding this, in precedent studies, we hypothesized that a low early-life glutathione level could serve as a redox signal programming a compensatory phenotype throughout life that might avoid the loss of evolutionary fitness (i.e., Romero-Haro and Alonso-Alvarez 2015; see also Romero-Haro et al. 2015; Romero-Haro et al. 2016). The question, then, arises about why that compensatory response was performed only by females.

An unknown sex-specific physiological mechanism would explain the findings. We should first address whether males and females differ in the values of both parameters. Regarding glutathione, adult females showed higher total glutathione levels than males (i.e., Romero-Haro and Alonso-Alvarez 2015, also in the present subsample: Estimate [males] = −0.167, SE = 0.083, *χ*^2^ = 4.154, *P* = 0.042). This result agrees with several studies in rodents and humans (Wang et al. 2003; Liu et al. 2004; Tarry-Adkins et al. 2006; Wang et al. 2020) but not with a recent meta-analysis in avian species (Vincze et al. 2022). On the other hand, in humans, women show longer telomeres than men (meta-analysis in Gardner et al. 2014). However, no clear difference was found in other vertebrates, including birds (meta-analysis in Remot et al. 2020). Here, zebra finch females and males did not significantly differ in their erythrocyte telomere length at 14 or 100 days of age (Tables 1 and S2, S3, and S4).

As a potential mechanism explaining our female-specific results, we may suspect the involvement of some sexual hormone. Indeed, estrogens seem to influence both glutathione and the activity of enzymes involved in telomere maintenance. Estrogens own antioxidants properties, including the upregulation of glutathione levels and glutathione-related antioxidant enzymatic activity (e.g., Behl et al. 1997; Borras et al. 2003; Strehlow et al. 2003; Liu et al. 2004; Vina et al. 2006; Borras et al. 2021). Similarly, estrogens upregulate telomerase (Kyo et al. 1999; Vina and Borras 2010; Zhou et al. 2013). This enzyme is critical in replenishing the telomeric DNA sequence shortened during cell division, preventing telomere attrition (e.g., Shay and Wright 2019). Low estrogen levels in women have also been associated with shorter telomeres (Shin and Lee 2016), supporting the link between sexual steroids and telomere length.

Here, we hypothesize that high oxidative stress derived from decreased glutathione levels during growth (described in Romero-Haro and Alonso-Alvarez 2015) may have favored the attainment of longer adult telomeres in BSO-treated females, probably by involving telomerase. It is well-established that oxidative stress can activate the Nuclear Factor (Erythroid-Derived 2)-Like 2 (NRF2) in vertebrates, including birds (e.g., Raghunath et al. 2018; Castiglione et al. 2020; Calabrese and Kozumbo 2021). For instance, exposure to moderate levels of pro-oxidant molecules under *in vitro* conditions activates the NRF2 pathway (Pi et al. 2008; Woods et al. 2009; Ma 2013; Calabrese and Kozumbo 2021 and references therein). In addition, BSO administration under *in vitro* or *in vivo* conditions induces NRF2 activation in different cell types of rodents (Lee et al. 2008; Valdovinos-Flores et al. 2019). NRF2 is considered the master regulator of inducible antioxidant responses, activating the enzyme in charge of glutathione synthesis (i.e., GCL; Wild et al. 1999) and telomerase-related genes (Ahmad et al. 2016; Yilmaz et al. 2022 and references therein). Hence, low glutathione levels could have activated NRF2. Then, NRF2 would have upregulated telomerase and, in consequence, produced longer telomeres.

That hypothetical NRF2-based hormetic mechanism could be sex specific. A lack of estrogens or inhibition of estrogen receptors reduced NRF2 gene expression in the gastric muscle cells of mice (Sprouse et al. 2020). This result may imply that low estrogen levels in males might limit their capacity for mounting compensatory responses. Similarly, estrogen replacement in menopausal women increased the expression of a gene involved in telomere protection (telomeric repeat-binding factor 2; TERF2) and restored total glutathione levels in blood decreased during climacteric (Borras et al. 2021). These studies suggest a physiological link between sex-specific endocrinology, glutathione, and telomere length that could explain our results. Unfortunately, the small blood volume available in this small bird and other technical limitations prevented quantifying telomerase activity and circulating hormones, which might have clarified the underlying mechanism.

We should also note that the apparent positive influence of nestling glutathione values on adult telomeres of control individuals (Fig. 2) would disagree with the positive impact of BSO-decreased glutathione levels on the same telomere measure in females. Nevertheless, the link to longevity could reconcile both findings. In control females, those who endured low glutathione levels early in life would have lived longer than other birds if they were able to generate long telomeres in adulthood (Fig. 3). This suggests that the ability to mount a hormetic response triggered by naturally low glutathione levels could improve fitness via longevity. However, this effect on longevity was not found in females whose glutathione concentrations were experimentally decreased. Perhaps, the BSO treatment imbalanced other physiological parameters (Romero-Haro and Alonso-Alvarez 2015) that indirectly affected survival, reducing any link to fitness. The loss of integration between antioxidants and oxidative stress-related parameter dynamics has been associated with physiological and even social costs, probably affecting individual fitness (Costantini et al. 2013; Fialkowski et al. 2022).

Finally, we must recognize that our longevity data were probably subject to measurement error due to logistic limitations inherent to the long-lasting monitoring (almost nine years long). Hence, the acceptance of the null hypothesis of an absence of significant longevity effects of the assessed variables and treatment should be taken with caution. Alternatively, the presence of weak or absent negative effects of the BSO treatment on fitness traits could also have been due to the proposed compensatory mechanism evening out the variability between treatment groups. Indeed, glutathione is only one piece of the antioxidant system and other antioxidants could have been upregulated to compensate BSO treatment. In conclusion, our experimental and correlational results support a link between early-life glutathione levels and adult telomere length in female birds that might influence their longevity to some extent. Now, more effort should be made to disentangle the proximate mechanisms involved in the physiological correlation of these two traits and potential implications in terms of evolutionary life history trade-offs.

## Supplementary Material

obad034_Supplemental_Files

## Data Availability

The data underlying this article are available in the article and in its online supplementary material.
