# Supplementary material for: Low Antioxidant Glutathione Levels Lead to Longer Telomeres: A Sex-Specific Link to Longevity?
Source: Integr Org Biol. 2023 Sep 23;5(1):obad034. doi: 10.1093/iob/obad034 (PMC10519275; doi:10.1093/iob/obad034)
Supplement: obad034_Supplemental_Files [file obad034_supplemental_files.zip › Supplementary material.pdf]

Supplementary Material for:

Low antioxidant glutathione levels lead to longer telomeres: a sex-specific link to longevity?

Experimental chronogram

Figure S1: Chronogram of the glutathione manipulation. BSO = buthionine sulfoximine. From (Romero-Haro and Alonso-Alvarez 2015).

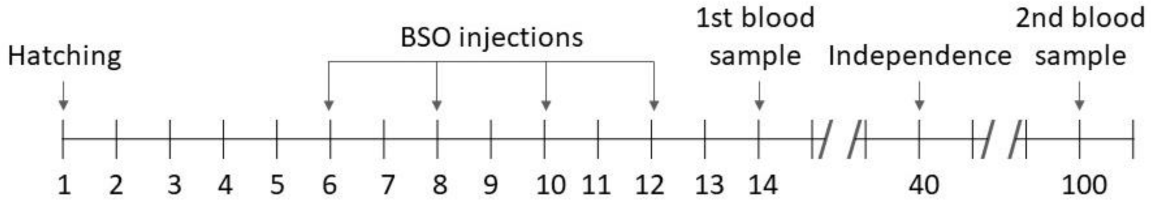

DNA extraction protocol

The protocol is based on the technology of magnetic beads and is implemented in the robotic platform EVO 100 (Tecan). With this technology, the nucleic acids attach to paramagnetic beads (SPeedBeads™ magnetic carboxylate modified particles GE Healthcare, 65152105050250) in the presence of salts, while sample contaminants are washed away during the process. The elution of DNA is done in low salinity conditions while the beads are retained through the use of magnets.

The detailed protocol includes the following steps: 315 µl of Ampure is added to a sample digestion volume of 315 µl. After mixing and allowing the mix to settle for five minutes the tubes are transferred to a magnet. After five minutes, the liquid is removed, keeping the magnetic beads (with the nucleic acids) that are adhered to the walls of the tube. The beads were washed with 900 µl of 80% ethanol, after 30 seconds, the liquid was removed, and the washing with ethanol was repeated. After removing the ethanol, the samples were allowed to dry for 10 minutes. For the elution of the nucleic acid,s the tubes were removed from the magnet and the

samples were eluted with 50 µl of TRIS Low EDTA (TLE). After 10 minutes of shaking at 60 ºC, the tubes were placed again in the magnet, and the liquid phase with the nucleic acids was pipetted in a new tube. The elution process was repeated again with 50 µl of TLE.

Supplementary statistical analyses

Body mass and age or body mass increase from the beginning of the experiment until 100 days were included as additional explanatory variables into the main text mixed effect model testing the effects of the oxidative stress treatment during development, sex, the level of glutathione at 14 days and their interaction on telomere length at adulthood (100 days). The brood identity nested into the cage identity and the laboratory session (plate) were included as random effects.

Second, we a ran linear mixed effect model to test for effects of the oxidative stress treatment during development, sex, the level of glutathione at 14 days (the manipulated variable) and their interaction on telomere length at 14 days (i.e.: shortly after the treatment). The brood identity nested into the cage identity and the laboratory session (plate) were included as random effects. Note that since the experimental treatment doses were administrated between days 4 and 12 after hatching, the telomere length at 14 days is already a post-treatment measurement and not a baseline value. Body mass and age, as well as body mass increase from the beginning of the experiment until 14 days, were included as explanatory variables in alternative models.

Finally, to test for a sex- or treatment-specific change in telomere length between days 14 and 100, we ran a (repeated-measurement) linear mixed effect model. The early treatment, sex, life stage (fledgling 14d / adult 100d) and their three-level interaction were included as explanatory variables and the ID of the brood nested into the ID of the origin cage, laboratory session, and the individual ID as random factors.

All statistical analyses were performed in *R* version 4.2.0. (R Core Team 2021). The *R* package *lme4* (v. 1.1.32) (Bates et al. 2015) was used to perform mixed effect models. The predictors' significance was obtained with ANOVA function from the *car* package (v. 3.1.1.) to

perform likelihood-ratio tests (Fox and Weisberg 2019). Normality and homoscedasticity assumptions of residuals were met.

Supplementary Results

Table S1: Repeatability of telomere measurements at 14 and 100 days when different fixed effects are included in the model. The identity of the individuals was fitted as a random factor in all the models.

Fixed effects in the model	R	SE	CI (95%)
Stage (i.e., fledging/adult)	0.435	0.064	0.301 – 0.558
Treatment	0.421	0.062	0.292 – 0.535
Sex	0.386	0.065	0.255 – 0.507
Stage + Treatment	0.437	0.063	0.309 – 0.548
Stage + Sex	0.402	0.063	0.274 – 0.524
Treatment + Sex	0.388	0.065	0.258 – 0.521
Stage + Treatment + Sex	0.404	0.066	0.267 – 0.531

Table S2: Effects of the early treatment, sex, glutathione levels at 14 days and their interaction on **telomere length at 100 days of life**. Body mass and age (model 1), body mass change (model 2) and telomere length at 14 days (model 3) were alternatively included. Nor body mass, body mass change, age or telomere length at 14 days affect the main text results.

**MODEL 1**					
**Explanatory variables**	**Estimate**	**SE**	χ^2	**df**	***P***
Intercept	0.047	0.746			
Treatment (Control)	-0.310	0.143	0.830	1	0.362
Sex (Males)	-0.405	0.160	2.289	1	0.130
14d Glutathione	-0.425	0.168	0.001	1	0.974
Body mass at 100 days	0.061	0.036	2.870	1	0.090
Age	-0.005	0.005	1.082	1	0.298
Treatment (Control) * Sex (Males)	0.454	0.205	5.046	1	**0.025**
Treatment (Control) * 14d Glutathione	1.041	0.274	11.791	1	**<0.001**
Sex (Males) * 14d Glutathione	0.339	0.265	0.003	1	0.955
Treatment (Control) * Sex (Males) * 14d Glutathione	-0.770	0.416	3.427	1	0.064
**Random factors**	**Variance**	**SE**			
Cage ID (Brood ID)	0.089	0.299			
Plate	0.557	0.747			
Residuals	0.380	0.617			
**MODEL 2**					
**Explanatory variables**	**Estimate**	**SE**	χ^2	**df**	***P***
Intercept	-0.122	0.394			
Treatment (Control)	-0.307	0.143	0.681	1	0.409
Sex (Males)	-0.392	0.160	1.895	1	0.169
14d Glutathione	-0.413	0.168	0.006	1	0.939
Body mass increase from 4 to 100 days	0.052	0.033	2.491	1	0.114
Treatment (Control) * Sex (Males)	0.463	0.205	5.240	1	**0.022**
Treatment (Control) * 14d Glutathione	1.020	0.274	11.210	1	**<0.001**
Sex (Males) * 14d Glutathione	0.309	0.266	0.004	1	0.950
Treatment (Control) * Sex (Males) * 14d Glutathione	-0.756	0.416	3.300	1	0.069
**Random factors**	**Variance**	**SE**			
Cage ID (Brood ID)	0.090	0.301			
Plate	0.539	0.735			
Residuals	0.381	0.617			
**MODEL 3**					
**Explanatory variables**	**Estimate**	**SE**	χ^2	**df**	***P***
Intercept	0.375	0.216			
Treatment (Control)	-0.321	0.138	0.436	1	0.509
Sex (Males)	-0.523	0.158	4.708	1	0.030
14d Glutathione	-0.510	0.170	0.120	1	0.729
Telomere length at 14d	0.164	0.064	6.611	1	**0.010**
Treatment (Control) * Sex (Males)	0.526	0.200	7.199	1	**0.007**
Treatment (Control) * 14d Glutathione	1.059	0.267	12.974	1	**<0.001**

Sex (Males) * 14d Glutathione	0.408	0.264	0.112	1	0.738
Treatment (Control) * Sex (Males) * 14d Glutathione	-0.780	0.409	3.637	1	0.057
**Random factors**	**Variance**	**SE**			
Cage ID (Brood ID)	0.101	0.317			
Plate	0.363	0.602			
Residuals	0.345	0.587			

Table S3: Effects of the early treatment, sex, glutathione levels at 14 days and their interaction on **telomere length at 14 days of life**. Body mass and age and body mass increase were alternatively included. Although the three-level interaction showed a slight trend to affect telomere length at 14 days, levels of glutathione at 14 days were not associated with telomere length at that age in either BSO or control males or females (all *P*-values > 0.081).

**MODEL 1**					
**Explanatory variables**	**Estimate**	**SE**	χ^2	**df**	***P***
Intercept	-0.235	0.372			
Treatment (Control)	-0.127	0.139	0.006	1	0.936
Sex (Males)	-0.021	0.168	0.586	1	0.444
14d Glutathione	0.265	0.175	0.897	1	0.343
Treatment (Control) * Sex (Males)	0.245	0.207	1.338	1	0.247
Treatment (Control) * 14d Glutathione	-0.183	0.282	0.547	1	0.460
Sex (Males) * 14d Glutathione	-0.556	0.274	1.007	1	0.316
Treatment (Control) * Sex (Males) * 14d Glutathione	0.826	0.441	3.516	1	0.061
**Random factors**	**Variance**	**SE**			
Cage ID (Brood ID)	0.360	0.600			
Plate	1.225	1.107			
Residuals	0.304	0.551			
**MODEL 2**					
**Explanatory variables**	**Estimate**	**SE**	χ^2	**df**	***P***
Intercept	0.086	0.988			
Treatment (Control)	-0.157	0.140	0.003	1	0.955
Sex (Males)	-0.057	0.169	0.536	1	0.464
14d Glutathione	0.215	0.177	0.295	1	0.587
Body mass at 14 days	0.019	0.049	0.145	1	0.703
Age	-0.036	0.043	0.679	1	0.410
Treatment (Control) * Sex (Males)	0.304	0.209	2.082	1	0.149
Treatment (Control) * 14d Glutathione	-0.159	0.282	0.524	1	0.469
Sex (Males) * 14d Glutathione	-0.512	0.274	0.841	1	0.359
Treatment (Control) * Sex (Males) * 14d Glutathione	0.764	0.442	2.991	1	0.084
**Random factors**	**Variance**	**SE**			
Cage ID (Brood ID)	0.353	0.594			
Plate	1.283	1.133			
Residuals	0.304	0.551			
**MODEL 3**					
**Explanatory variables**	**Estimate**	**SE**	χ^2	**df**	***P***
Intercept	-0.321	0.446			
Treatment (Control)	-0.159	0.139	<0.001	1	0.996
Sex (Males)	-0.055	0.169	0.510	1	0.475
14d Glutathione	0.228	0.176	0.506	1	0.477
Body mass increase from 4 to 14 days	0.019	0.041	0.209	1	0.648
Treatment (Control) * Sex (Males)	0.297	0.209	1.993	1	0.158
Treatment (Control) * 14d Glutathione	-0.159	0.281	0.597	1	0.440

Sex (Males) * 14d Glutathione	-0.522	0.273	0.859	1	0.354
Treatment (Control) * Sex (Males) * 14d Glutathione	0.782	0.439	3.175	1	0.075
**Random factors**	**Variance**	**SE**			
Cage ID (Brood ID)	0.362	0.602			
Plate	1.288	1.135			
Residuals	0.299	0.547			

Table S4: Effects of the early treatment, sex, life stage (fledging vs adult) and their interaction on **telomere length measured at two stages (Fledging 14d old and adulthood 100d old).** Similar to the main text model of telomere length at 100 days, the early treatment and sex showed an interaction on telomere length. Although *post hoc* contrast did not reveal any significant comparison, the telomere length of BSO females was longer than the telomeres of the rest of the groups. Besides, the sex and life stage also had a significant interaction effect on telomere length. Among females, telomere elongated from 14 to 100 days (mean ± 1SE: -0.168 ± 0.219 and 0.251 ± 0.218, respectively; t = 3.905, df = 174, P < 0.001), but telomere length did not significantly change with age among males (14d: 0.012 ± 0.222; 100d: 0.082 ± 0.221; t = 0.639, df = 171, P = 0.524). The (non-significant) three-level interaction is represented in figure S2.

**Explanatory variables**	**Estimate**	**SE**	χ^2	**df**	***P***
Intercept	0.481	0.229			
Treatment (Control)	-0.459	0.160	0.034	1	0.854
Sex (Males)	-0.478	0.173	0.002	1	0.966
Life stage (Fledging)	-0.688	0.146	11.206	1	**0.001**
Treatment (Control) * Sex (Males)	0.618	0.230	4.271	1	**0.039**
Treatment (Control) * Life stage (Fledging)	0.538	0.213	3.389	1	0.066
Sex (Males) * Stage (Fledging)	0.612	0.219	4.987	1	**0.026**
Treatment (Control) * Sex (Males) * Life stage (Fledging)	-0.526	0.305	2.972	1	0.085
**Random factors**	**Variance**	**SE**			
Cage ID (Brood ID)	0.112	0.334			
Plate	0.390	0.624			
Individuals ID	0.090	0.301			
Residuals	0.502	0.708			

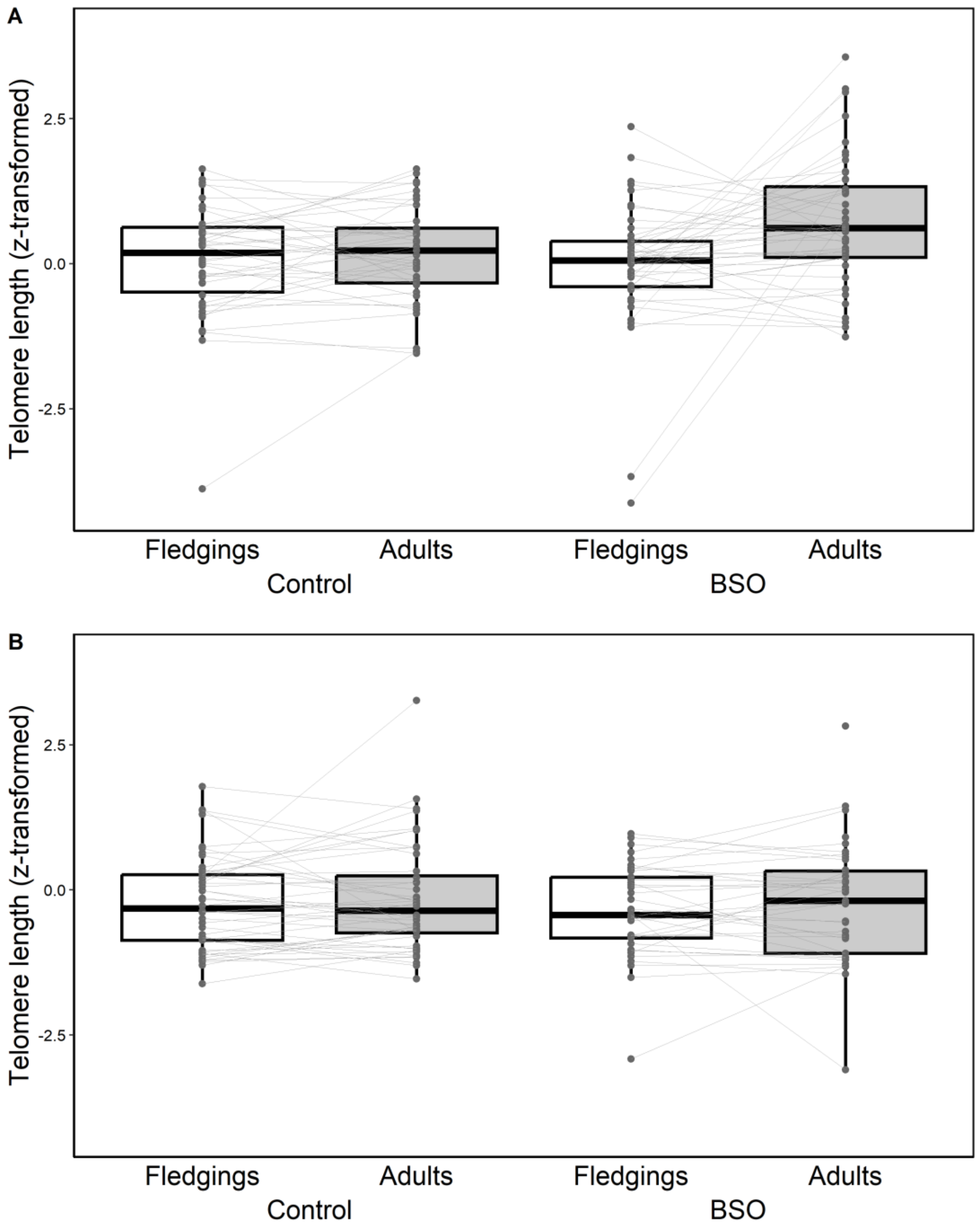

Figure S2: Telomere dynamic between 14 and 100 days among control and BSO A) females and B) males. Telomere length values (T/S ratio) were sqrt- and z-transformed (standardized). Horizontal lines in boxplots represent means and interquartile ranges. Individual data points are shown and those at 14 and 100 days from the same individual are connected with light grey lines. Note that telomere length at 14 days is a post-treatment measurement, not a baseline one. Among BSO females, telomeres elongated from 14 to 100 days (mean ± 1SE: -0.207 ± 0.231 and 0.481 ± 0.230, respectively; t = 4.695, df = 175, P < 0.001). Telomere length did not significantly change with age among control females (14d: -0.128 ± 0.236; 100d: 0.022 ± 0.235; t = 0.961, df = 171, P = 0.338), BSO males (14d: -0.073 ± 0.240; 100d: 0.002 ± 0.238; t = 0.465, df = 173, P = 0.643) or control males (14d: 0.098 ± 0.233; 100d: 0.161 ± 0.233; t = 0.439, df = 168, P = 0.661).

Table S5: Effects of the early treatment, sex, levels of glutathione at 14d-old (the manipulated variable) and their interaction on **telomere length at 100 days of life** when removing the lowest telomere length value (one BSO male). Results are similar to those reported in the main text.

**Explanatory variables**	**Estimate**	**SE**	χ^2	**df**	***P***
Intercept	0.332	0.254			
Treatment (Control)	-0.325	0.134	1.960	1	0.162
Sex (Males)	-0.279	0.153	0.399	1	0.528
14d Glutathione	-0.369	0.157	0.047	1	0.829
Treatment (Control) * Sex (Males)	0.412	0.193	4.670	1	**0.031**
Treatment (Control) * 14d Glutathione	0.950	0.257	11.564	1	**<0.001**
Sex (Males) * 14d Glutathione	0.288	0.249	<0.001	1	0.989
Treatment (Control) * Sex (Males) * 14d Glutathione	-0.672	0.393	2.934	1	0.087
**Random factors**	**Variance**	**SE**			
Cage ID (Brood ID)	0.122	0.349			
Plate	0.541	0.736			
Residuals	0.318	0.564			

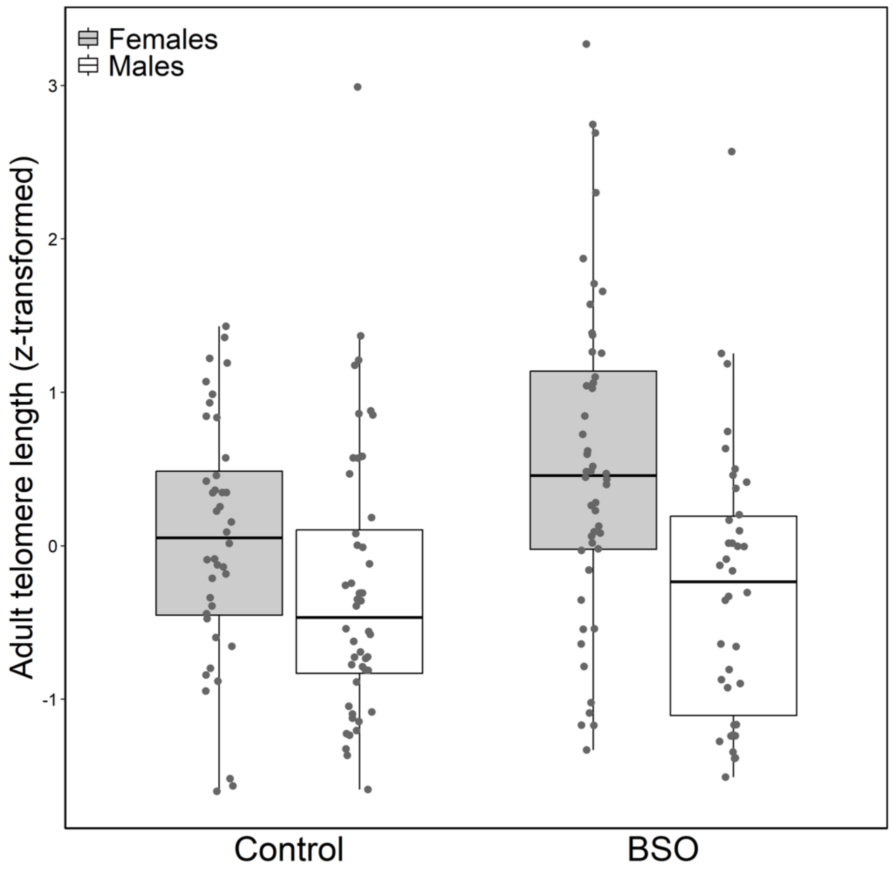

Figure S3: Telomere length at 100 days among Control and BSO individuals when removing the lowest telomere length value (one BSO male, see table S4). Telomere length values (T/S ratio) were sqrt- and z-transformed. Horizontal lines in boxplots represent means and interquartile ranges. Individual data points are shown.

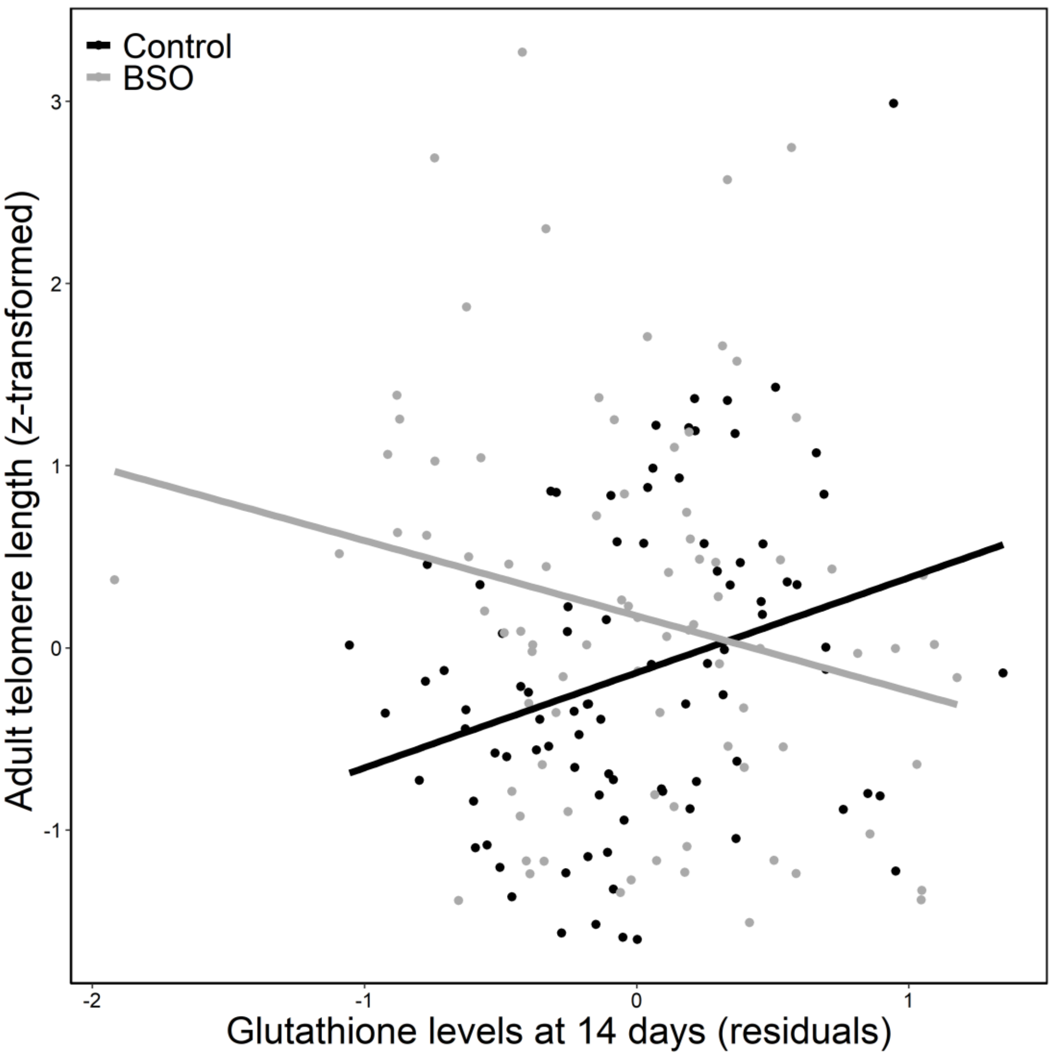

Figure S4: Association between telomere length at 100 days and glutathione levels at 14 days among control (in black) and BSO (in grey) individuals when removing the lowest telomere length value. Glutathione levels are the residuals obtained from mixed effect models with the ID of the brood nested into the ID of the origin cage and the laboratory session as random effects and the early treatment as an explanatory variable. Telomere length values (T/S ratio) were sqrt- and z-transformed. Note that association is positive and significant among control individuals (Estimate = 0.324, SE = 0.132, X^2 = 6.039, P = 0.014) and not significant among BSO individuals (Estimate = -0.253, SE = 0.131, X^2 = 3.700, P = 0.055). When additionally removing the lowest value of glutathione of one BSO individual, the results of table S4 did not change and the association between telomere length at 100 days and glutathione levels at 14 days among BSO individuals did not get significance (Estimate = -0.283, SE = 0.146, X^2 = 3.749, P = 0.053).

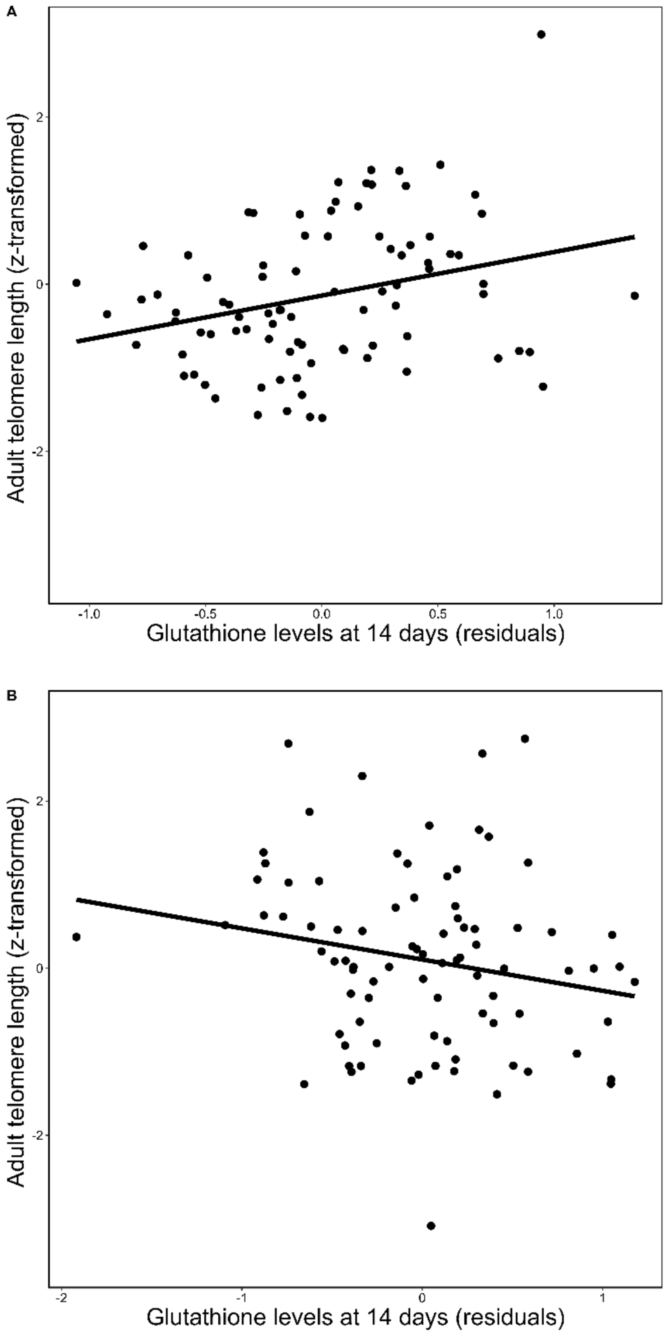

Figure S5: Association between telomere length at 100 days and glutathione levels at 14 days among A) control and B) BSO individuals. Glutathione levels are the residuals obtained from mixed effect models with the ID of the brood nested into the ID of the origin cage and the laboratory session as random effects and the early treatment as an explanatory variable. Telomere length values (T/S ratio) were sqrt- and z-transformed. Note that association is significant among control individuals (panel A) and non-significant among BSO individuals (panel B).

Table S6: Effects of the early treatment, sex, telomere length at adulthood and their interaction on individuals' **reproductive output**.

**Latency to the first reproduction**					
**Explanatory variables**	**Estimate**	**SE**	χ^2	**df**	***P***
Intercept	88.655	7.825			
Treatment (Control)	9.717	10.106	0.583	1	0.445
Sex (Males)	1.235	10.129	0.306	1	0.580
Telomere length at 100d	1.474	10.070	0.826	1	0.363
Releasing event (second)	-12.398	7.036	3.105	1	0.078
Treatment (Control) * Sex (Males)	-9.133	14.170	0.413	1	0.520
Treatment (Control) * Telomere length at 100d	-8.210	17.371	0.481	1	0.488
Sex (Males) * Telomere length at 100d	16.232	15.496	1.630	1	0.202
Treatment (Control) * Sex (Males) * Telomere length at 100d	-1.288	25.457	0.003	1	0.960
**Number of eggs**					
**Explanatory variables**	**Estimate**	**SE**	χ^2	**df**	***P***
Intercept	1.441	0.074			
Treatment (Control)	0.040	0.109	0.039	1	0.844
Sex (Males)	0.057	0.109	0.002	1	0.966
Telomere length at 100d	-0.004	0.110	0.058	1	0.810
Treatment (Control) * Sex (Males)	-0.106	0.153	0.479	1	0.490
Treatment (Control) * Telomere length at 100d	0.056	0.187	0.109	1	0.742
Sex (Males) * Telomere length at 100d	0.007	0.166	<0.001	1	0.989
Treatment (Control) * Sex (Males) * Telomere length at 100d	-0.023	0.274	0.007	1	0.933
**Hatching success**					
**Explanatory variables**	**Estimate**	**SE**	χ^2	**df**	***P***
Intercept	0.783	0.183			
Treatment (Control)	-0.081	0.250	0.462	1	0.497
Sex (Males)	0.063	0.263	0.009	1	0.923
Telomere length at 100d	0.186	0.254	0.244	1	0.621
Treatment (Control) * Sex (Males)	-0.098	0.358	0.074	1	0.786
Treatment (Control) * Telomere length at 100d	-0.445	0.442	2.884	1	0.089
Sex (Males) * Telomere length at 100d	-0.141	0.384	0.542	1	0.462
Treatment (Control) * Sex (Males) * Telomere length at 100d	-0.243	0.640	0.144	1	0.704

Table S7: Effects of the telomere length (at fledging stage, 14 days, or at adulthood, 100 days), early treatment, sex, and their interaction on individuals' **longevity**

**Telomere length at 14 days**					
**Explanatory variables**	**β**	**SE**	**χ^2**	**df**	***P***
Treatment (Control)	0.243	0.262	0.179	1	0.673
Sex (Males)	-0.138	0.275	2.760	1	0.097
Telomere length at 14d	0.109	0.430	0.112	1	0.737
Treatment (Control) * Sex (Males)	-0.341	0.378	0.815	1	0.367
Treatment (Control) * Telomere length at 14d	-0.262	0.769	0.428	1	0.513
Sex (Males) * Telomere length at 14d	0.472	0.808	0.409	1	0.522
Treatment (Control) * Sex (Males) * Telomere length at 14d	-0.229	0.796	0.044	1	0.833
**Telomere length at 100 days**					
**Explanatory variables**	**β**	**SE**	**χ^2**	**df**	***P***
Treatment (Control)	0.200	0.277	0.354	1	0.552
Sex (Males)	-0.144	0.273	2.322	1	0.128
Telomere length at 100d	0.416	0.254	0.452	1	0.502
Treatment (Control) * Sex (Males)	-0.233	0.386	0.457	1	0.499
Treatment (Control) * Telomere length at 100d	-0.961	0.527	1.492	1	0.222
Sex (Males) * Telomere length at 100d	-0.391	0.377	0.127	1	0.722
Treatment (Control) * Sex (Males) * Telomere length at 100d	0.955	0.705	1.833	1	0.176

Table S8: Effects of the sex, early glutathione levels and adult telomere length and their interactions on the **longevity of control birds**. BSO individuals were not included in this analysis since glutathione and telomere values were not correlated in this group.

**Explanatory variables**	**β**	**SE**	**χ^2**	**df**	***P***
Sex (Males)	-0.266	0.293	3.181	1	0.075
14d Glutathione	0.759	0.436	4.495	1	**0.034**
Telomere length at 100d	-0.725	0.452	1.452	1	0.228
Sex (Males) * 14d Glutathione	-0.173	0.600	1.065	1	0.302
Sex (Males) * Telomere length at 100d	1.025	0.677	0.876	1	0.349
14d Glutathione * Telomere length at 100d	2.390	0.982	0.254	1	0.613
Sex (Males) * 14d Glutathione * Telomere length at 100d	-3.337	1.251	7.112	1	**0.008**

Interaction between early glutathione levels and telomere length on longevity

We initially addressed the effect of a potential interaction between early glutathione values and adult telomere length on longevity of control individuals by testing a std-residual of telomere length on early glutathione levels in a mixed Cox model (Coxme R package; see also Statistical Analysis in the main text). The residual was obtained by a precedent mixed model testing the effect of early glutathione level (covariate) on adult telomere length (dependent variable). The latter model included the brood identity nested into the identity of the origin cage and the telomere laboratory session as random terms. The residual between these parameters or its interaction with the sex did not explain longevity (all $P > 0.10$). Alternative to this approach, we directly tested the simultaneous effect of these parameters and their interaction on longevity. Such an approach avoids an intermediate step (extracting residuals and then using them in another model) and does not assume the linearity of the covariation. In this case, the effect of the interactive covariates did significantly influence longevity among control individuals depending on the sex (Table S8 and main text).

References

Bates D, Mächler M, Bolker B, Walker S. 2015. Fitting Linear Mixed-Effects Models Using lme4. Journal of Statistical Software 67(1):1 - 48.

Fox J, Weisberg S. 2019. An R Companion to Applied Regression Sage.

R Core Team. 2021. R: A Language and Environment for Statistical Computing. Vienna, Austria: R Foundation for Statistical Computing.

Romero-Haro AA, Alonso-Alvarez C. 2015. The level of an intracellular antioxidant during development determines the adult phenotype in a bird species: a potential organizer role for glutathione. Am Nat 185(3):390-405.
